# Awareness of Thyroid Diseases and Their Risk Factors Among the Residents of Jeddah, Saudi Arabia

**DOI:** 10.7759/cureus.62113

**Published:** 2024-06-10

**Authors:** Randa M Alharazi, Lulu K Almarri, Heba M Ibrahim, Lama S Abdulshakour, Mashael A Ahmad, Rahaf A Bashool

**Affiliations:** 1 Internal Medicine, Ibn Sina National College for Medical Studies, Jeddah, SAU; 2 Medicine, Ibn Sina National College for Medical Studies, Jeddah, SAU

**Keywords:** saudi arabia, knowledge, attitude, awareness, thyroid diseases

## Abstract

Background

The thyroid gland is responsible for regulating many aspects of body functions. Despite their global prevalence, thyroid disorders often go underdiagnosed, which can lead to serious health complications. In Saudi Arabia, the overall prevalence was 49.76%, among which subclinical hypothyroidism was the most prevalent type. Raising awareness and knowledge about thyroid diseases and their risk factors is essential for the prevention and early treatment of these disorders.

Aim and objectives

To assess the awareness of thyroid diseases and their risk factors among the residents of Jeddah, Saudi Arabia, as well as understand their attitudes and underlying influencing factors toward thyroid health.

Methods

A cross-sectional study was conducted in Jeddah, Saudi Arabia, from January 2023 to December 2023. The study included a diverse sample of Saudi and non-Saudi participants aged 18 to 65 years. A self-administered online questionnaire translated into Arabic was used to collect information.

Results

The study involved 393 participants, 72.5% female and 27.5% male. Most participants held a bachelor's degree or higher (78.1%). Hypothyroidism was the most prevalent diagnosed thyroid disease (14.0%). Only 20% of participants had good knowledge. Respondents were relatively less aware of the risks associated with pregnancy and the postpartum period (35%), medications such as amiodarone (26%), eating soya beans (22%), and gastrointestinal tract (GIT) symptoms of thyroid diseases (36%). Attitudes toward thyroid health were generally negative (85.5%). However, a significant association was noted between a history of thyroid disease and a positive attitude (p = 0.002). Educational level and employment status were strong determinants of knowledge levels (p = 0.036 and 0.005, respectively). A positive correlation was found between knowledge levels and attitudes (r = 0.321, p < 0.001).

Conclusion

The study showed a low level of awareness among participants living in Jeddah, especially the unemployed and those with low levels of education. Their unawareness of the possible risks of thyroid diseases during pregnancy should be thoroughly addressed by public campaigns.

## Introduction

The thyroid is a gland that secretes triiodothyronine (T3) and thyroxin hormone (T4), which is responsible for bone formation, cardiac contractility, protein synthesis, and regulating the basal metabolic rate of the body. Moreover, thyroid hormones play a significant role in the neurological and mental development of children [1]. Thyroid disease is one of the most common pathologies in the world, with two of the most clinically important subgroups being iodine deficiency and thyroid goiter and thyroid cancer. Thyroid disorders are considered among the diseases that can be anticipated and avoided before they present clinically [2]. Thus, prevention, early diagnosis, and prompt treatment are fundamental steps to reduce healthcare costs related to thyroid disorders [2,3]. Thyroid illness is caused by a deficiency in iodine or by autoimmune diseases [4,5]. Other studies have shown that thyroid disease is caused by inflammation or particular medical operations such as radiation, thyroid surgery, or by a hereditary factor [6].

Despite being one of the most common endocrine disorders, thyroid disorders are among the most underdiagnosed and neglected chronic health conditions globally [7,8]. Sixty percent of individuals with thyroid dysfunction are unaware of their condition worldwide [1]. Thyroid disorders can be easily missed or confused with other medical conditions because most of the symptoms are not specific [9]. However, if left untreated, thyroid disorders may lead to complications that may impact the patient's quality of life [10].

Thyroid disorders are one of the most common medical conditions worldwide [11]. In Saudi Arabia, the overall prevalence was 49.76%, among which subclinical hypothyroidism was the most prevalent type (39.25%), followed by primary hypothyroidism (5.3%) [6]. However, studies found that the prevalence of thyroid dysfunction varies by age, gender, race/ethnicity, geographical distribution, and amount of dietary iodine intake [6,12]. Another study on the risk factors for thyroid dysfunction among type 2 diabetic patients in Saudi Arabia indicated that the prevalence of different types of thyroid dysfunction was 28.5%, with 25.3% having hypothyroidism and 3.2% having hyperthyroidism. The hypothyroidism group showed that 15.8% have clinical hypothyroidism, while 9.5% have subclinical hypothyroidism [9].

Awareness of thyroid diseases among the population is essential for the prevention, early detection, and proper treatment of thyroid diseases. In a previous study in the Eastern Province, of Saudi Arabia, there is a general lack of knowledge about thyroid diseases and their risk factors [5]. In a systematic review by Li et al. [13] about the knowledge, awareness, and perception toward thyroid cancer in the general population, the authors found that both the general population and medical students have relatively poor levels of knowledge and perception of thyroid cancer and risk factors. Most participants are increasingly turning to the Internet and social media to obtain information about thyroid cancer. Moreover, this study indicated that poor levels of knowledge were strongly related to educational attainment and the type of participants. Hence, they concluded that encouraging intervention providers to conduct health promotion campaigns to enhance the knowledge and awareness of thyroid cancer helps in the prevention and early diagnosis of the disease [13]. There is a limited number of studies in Saudi Arabia that assess the awareness of thyroid diseases, especially among the population of the Western region.

Therefore, we aimed to evaluate the knowledge of thyroid disease and its risk factors among Jeddah residents and understand their attitudes toward thyroid health. Moreover, we aimed to identify underlying factors about the knowledge and attitude of participants.

## Materials and methods

Study design and participants

A cross-sectional study was conducted among the residents of Jeddah, Saudi Arabia, population from January 2023 to December 2023. The representative sample included Saudi and non-Saudi males and females of different age groups living in Jeddah, Saudi Arabia. Participants under 18 years, people working in healthcare settings, residents other than Jeddah, and those who declined to participate were excluded.

Sample size calculation

The sample size was calculated using QuestionPro (https://www.questionpro.com/sample-size-calculator/), assuming that the total targeted population is 3,751,722 individuals and the calculated sample size required is 385 individuals. Taking into consideration a 95% confidence interval and 5% margin of error. Participants were included using a convenient nonrandomized technique.

Data collection and tools

A questionnaire translated into Arabic using the Google questionnaire platform was disseminated online to each participant through social media to gather information on the following variables divided into three sections: (1) sociodemographics and past medical history of thyroid disorders; (2) knowledge of risk factors of thyroid diseases, the clinical picture of thyroid diseases, and their prevention (this section contains 19 questions, and each answer may be “yes” (scored 1), “no,” or “I do not know” (scored 0)); (3) attitude of residents about thyroid diseases containing six questions scored from 1 to 5 and other three questions that may have more than one choice not included in the score. As regards the distribution of level of knowledge among participants, we considered that getting a score of less than 10/19 (<50%) as having poor knowledge, form 10-15 (50-75%) as fair knowledge, and more than 15 (>75%) as good knowledge. Moreover, we considered participants who have a score of 23/30 or more (75%) as having positive attitudes toward thyroid disease.

Participants were informed that participation is completely voluntary. Consent was obtained from each participant before research, no name was recorded on the questionnaires, and all the personal information of participants was kept confidential. The questionnaire was an adaptation of the questionnaire developed by Alyahya et al. [5]. The questionnaire was revised by two endocrine expert consultants to check its clarity and content validity. Moreover, a pilot study was conducted on around 10% of the sample size (30 participants) before data collection and the Cronbach alpha test result was 0.735, which is considered reliable.

Statistical analysis

Data were analyzed using Statistical Product and Service Solutions (SPSS, version 23; IBM SPSS Statistics for Windows, Armonk, NY). Descriptive statistics included median and interquartile ranges (IQRs) for continuous variables and numbers and percentages for categorical variables. Comparison between variables was performed using the "chi-square" test for categorical variables, and Student’s t-test and one-way ANOVA were used for quantitative variables. The level of significance of the study was set at 0.05.

## Results

A total of 393 participants were enrolled in this study. The median age of the participants was 30 years with an IQR of 21. Most of the participants were females (285; 72.5%) and Saudi (293; 74.6%), most had a higher educational level of bachelor’s or higher (307; 78.1%), and most did not have thyroid disease before (335; 85.2%). Among those who have been diagnosed, the most common type was hypothyroidism (55; 14.0%)), followed by hyperthyroidism (10; 2.5%), thyroid cancer (5; 1.3%), and thyroid nodules (6; 1.5%). Our results showed that 173 (44% of respondents) had a family member with a history of thyroid disease. Among those with a family history, hypothyroidism (107; 27.2%) was the most common type, followed by hyperthyroidism (31; 7.9%) and thyroid cancer (20; 5.1%) (Table 1).

**Table 1:** Demographic and clinical history of the participants IQR: interquartile range; SAR: Saudi Arabian Riyal

Sociodemographic data		Count	%
Gender	Female	285	72.5%
	Male	108	27.5%
Age, median IQR		30 (21)	
Nationality	Non-Saudi	100	25.4%
	Saudi	293	74.6%
Marital status	Unmarried	226	57.5%
	Married	167	42.5%
Educational level	High school or below	86	21.9%
	Bachelor or higher	307	78.1%
Employment	Student	136	34.6%
	Unemployed	98	24.9%
	Employed	159	40.5%
Annual income (in SAR)	<120,000	231	58.8%
	≥120,000	162	41.2%
Have you been diagnosed with thyroid disease before?	No	335	85.2%
	I do not know	0	0.0%
	Yes	58	14.8%
If you answered the previous questions with “yes,” what was the type of thyroid disease you were diagnosed with?	No previous thyroid disease	209	53.2%
	I do not know	108	27.5%
	Hyperthyroidism	10	2.5%
	Hypothyroidism	55	14.0%
	Thyroid nodules	6	1.5%
	Thyroid cancer	5	1.3%
Have any one of your family members had thyroid disease before?	No	220	56.0%
	I do not know	0	0.0%
	Yes	173	44.0%
If you answered the previous questions with yes, what was the type of thyroid diseases they have been diagnosed with?	Nothing	138	35.1%
	I do not know	91	23.2%
	Hyperthyroidism	31	7.9%
	Hypothyroidism	107	27.2%
	Thyroid nodules	6	1.5%
	Thyroid cancer	20	5.1%

Our results showed that 51.7% have poor knowledge and 28.2% have fair knowledge. Only 20.1% have good knowledge.

The general knowledge about thyroid diseases seems reasonably acceptable (median of 10, range of 19). However, there are certain areas where respondents show less certainty or awareness, such as the impact of pregnancy and postpartum (47.8%), medications such as amiodarone and lithium (62.8% and 65.1%, respectively), and eating soy food (59.8%) as a risk factor of thyroid diseases. Moreover, respondents are less knowledgeable about the gastrointestinal tract (GIT) symptoms of thyroid diseases (63.6%) (Table 2).

**Table 2:** Descriptive statistics of the overall knowledge questions among the participants

		Count	%
Do you think smoking is a risk factor for thyroid diseases?	No	80	20.4%
	I do not know	134	34.1%
	Yes	179	45.5%
Do you think radiation exposure is a risk factor for thyroid diseases?	No	48	12.2%
	I do not know	123	31.3%
	Yes	222	56.5%
Do you think insufficient or excess iodine intake is a risk factor for thyroid diseases?	No	30	7.6%
	I do not know	141	35.9%
	Yes	222	56.5%
Do you think females are more at risk of having thyroid diseases?	No	27	6.9%
	I do not know	103	26.2%
	Yes	263	66.9%
Do you think the pregnancy and postpartum period are risk factors for thyroid diseases?	No	64	16.3%
	I do not know	188	47.8%
	Yes	141	35.9%
Do you think the medication amiodarone (known commercially as Pacerone, Cordarone, Advadarone, and Sedacoron) is a risk factor for thyroid diseases?	No	44	11.2%
	I do not know	247	62.8%
	Yes	102	26.0%
Do you think lithium intake is a risk factor for thyroid disease?	No	34	8.7%
	I do not know	256	65.1%
	Yes	103	26.2%
Do you think autoimmune diseases can be associated with thyroid diseases?	No	46	11.7%
	I do not know	161	41.0%
	Yes	186	47.3%
Do you think thyroid diseases can lead to cancer?	No	44	11.2%
	I do not know	144	36.6%
	Yes	205	52.2%
Do you think feeling cold and weight gain are common symptoms of having hypothyroidism?	No	32	8.1%
	I do not know	70	17.8%
	Yes	291	74.0%
Do you think feeling hot and weight loss are common symptoms of having hyperthyroidism?	No	41	10.4%
	I do not know	82	20.9%
	Yes	270	68.7%
Do you think the neck lump can be a sign of thyroid diseases?	No	36	9.2%
	I do not know	78	19.8%
	Yes	279	71.0%
Do you think fatigue can be a symptom of thyroid diseases?	No	33	8.4%
	I do not know	96	24.4%
	Yes	264	67.2%
Do you think diarrhea, constipation, or stomachache can be symptoms of thyroid diseases?	No	99	25.2%
	I do not know	151	38.4%
	Yes	143	36.4%
Do you think skin and nail changes or hair loss can be signs of thyroid diseases?	No	47	12.0%
	I do not know	126	32.1%
	Yes	220	56.0%
Do you think bulging eyes can be a sign of thyroid diseases?	No	43	10.9%
	I do not know	130	33.1%
	Yes	220	56.0%
Do you think being away from soy food is one of ways to prevent thyroid diseases in women?	No	69	17.6%
	I do not know	235	59.8%
	Yes	89	22.6%
Do you think early thyroid function tests can prevent the complication of thyroid diseases?	No	16	4.1%
	I do not know	49	12.5%
	Yes	328	83.5%
Do you think a well-balanced diet is essential to prevent thyroid diseases?	No	30	7.6%
	I do not know	77	19.6%
	Yes	286	72.8%

Our results showed that 85.5% of the surveyed individuals have a negative attitude about thyroid diseases (median: 19, range: 19). More than 50% of respondents are unaware of their family history as a potential risk factor for thyroid diseases. Moreover, a significant percentage of respondents (70.8%) agree or strongly agree that it would be very serious if they got thyroid disease. However, our results showed that 87.8% of the participants will go to a health facility if they have a thyroid disease. Regarding knowledge sources about thyroid disease, combined sources, physicians, and social media (37.2%, 25.7%, and 14.5%, respectively) were the most common (Table 3).

**Table 3:** Descriptive statistics of the overall attitude questions among the participants

		Count	%
I can get thyroid disease.	Strongly disagree	35	8.9%
	Disagree	49	12.5%
	Neutral	152	38.7%
	Agree	103	26.2%
	Strongly agree	54	13.7%
My family history makes it more likely to get thyroid disease.	Strongly disagree	42	10.7%
	Disagree	89	22.6%
	Neutral	83	21.1%
	Agree	96	24.4%
	Strongly agree	83	21.1%
Having thyroid disease may limit my daily activities.	Strongly disagree	35	8.9%
	Disagree	80	20.4%
	Neutral	81	20.6%
	Agree	131	33.3%
	Strongly agree	66	16.8%
Having thyroid disease negatively impacts a person’s job performance.	Strongly disagree	38	9.7%
	Disagree	59	15.0%
	Neutral	97	24.7%
	Agree	133	33.8%
	Strongly agree	66	16.8%
I have to do a screening test for thyroid functions.	Strongly disagree	15	3.8%
	Disagree	19	4.8%
	Neutral	48	12.2%
	Agree	144	36.6%
	Strongly agree	167	42.5%
It would be very serious if I got thyroid disease	Strongly disagree	16	4.1%
	Disagree	18	4.6%
	Neutral	79	20.1%
	Agree	129	32.8%
	Strongly agree	151	38.4%
What will you do if you think that you have a thyroid disease?	Take iodine compounds	19	4.8%
	Go to a health facility	345	87.8%
	Use costus	16	4.1%
	Go to a traditional healer	13	3.3%
What is your source of knowledge about thyroid disease?	Physicians	101	25.7%
	Family members	35	8.9%
	Friends	22	5.6%
	Social media	57	14.5%
	Books and magazines	21	5.3%
	TV	11	2.8%
	Combined	146	37.2%

We also investigated and compared different knowledge levels among participants regarding their sociodemographic data. Educational level and employment status appear to be particularly strong determinants of knowledge. Participants with a high school education or below have a significantly higher percentage of "Poor" knowledge (62%) compared to those with a bachelor's degree or higher (p = 0.036). Unemployed participants have poor knowledge levels compared to employed who have fair knowledge levels. Moreover, students have the highest knowledge level among the participants (p = 0.005). However, having thyroid disease does not have a significant effect on knowledge level (p = 0.061) (Table 4).

**Table 4:** Comparison of different knowledge levels among participants regarding socio-demographic data *F Value; P <0.05 is significant; IQR: interquartile range; SAR: Saudi Arabian Riyal

		Poor knowledge (203; 51.7%)		Fair knowledge (111; 28.2%)		Good knowledge (79; 20.1%)		Chi square	P value
		Count	Percent	Count	Percent	Count	Percent		
Gender	Female	145	71.4%	85	76.6%	55	69.6%	1.37	505
	Male	58	28.6%	26	23.4%	24	30.4%		
Age, median (IQR)		32 (22)		32 (19)		24 (18)		1.43*	0.242
Nationality	Non-Saudi	53	26.1%	24	21.6%	23	29.1%	1.46	0.481
	Saudi	150	73.9%	87	78.4%	56	70.9%		
Residence area	Jeddah	151	74.4%	88	79.3%	66	83.5%	2.99	0.224
	Others	52	25.6%	23	20.7%	13	16.5%		
Marital status	Unmarried	112	55.2%	63	56.8%	51	64.6%	2.09	0.353
	Married	91	44.8%	48	43.2%	28	35.4%		
Educational level	High school or below	48	23.6%	29	26.1%	9	11.4%	6.62	0.036
	Bachelor or higher	155	76.4%	82	73.9%	70	88.6%		
Employment	Student	60	29.6%	36	32.4%	40	50.6%	14.92	0.005
	Unemployed	61	30.0%	27	24.3%	10	12.7%		
	Employed	82	40.4%	48	43.2%	29	36.7%		
Annual income (in SAR)	<120,000	117	57.6%	69	62.2%	45	57.0%	0.74	0.690
	≥120,000	86	42.4%	42	37.8%	34	43.0%		
History of thyroid disease	No	169	83.3%	92	82.9%	74	93.7%	5.59	0.061
	Yes	34	16.7%	19	17.1%	5	6.3%		

Our results showed that the only determinant of positive attitude is having previous thyroid disease when comparing participants’ attitudes regarding their sociodemographic data. This association is statistically significant (p = 0.002) (Table 5).

**Table 5:** Determinants of different attitudes among participants regarding sociodemographic data *F Value; P <0.05 is significant; IQR: interquartile range; SAR: Saudi Arabian Riyal

		Negative attitude (336; 85.5%)		Positive attitude (57; 14.5%)		Chi square	P value
		Count	Percent	Count	Percent		
Gender	Female	243	72.3%	42	73.7%	0.05	0.831
	Male	93	27.7%	15	26.3%		
Age, median (IQR), years		30 (20)		31 (23)		0.19*	0.492
Nationality	Non-Saudi	84	25.0%	16	28.1%	0.242	0.623
	Saudi	252	75.0%	41	71.9%		
Residence area	Jeddah	263	78.3%	42	73.7%	0.59	0.442
	Others	73	21.7%	15	26.3%		
Marital status	Unmarried	192	57.1%	34	59.6%	1.25	0.723
	Married	144	42.9%	23	40.4%		
Educational level	High school or below	73	21.7%	13	22.8%	0.033	0.855
	Bachelor or higher	263	78.3%	44	77.2%		
Employment	Student	114	33.9%	22	38.6%	0.49	0.785
	Unemployed	85	25.3%	13	22.8%		
	Employed	137	40.8%	22	38.6%		
Annual income (SAR)	<120,000	200	59.5%	31	54.4%	0.53	0.466
	≥120,000	136	40.5%	26	45.6%		
Past history of thyroid disease	No	294	87.5%	41	71.9%	9.39	0.002
	Yes	42	12.5%	16	28.1%		

**Figure 1:**
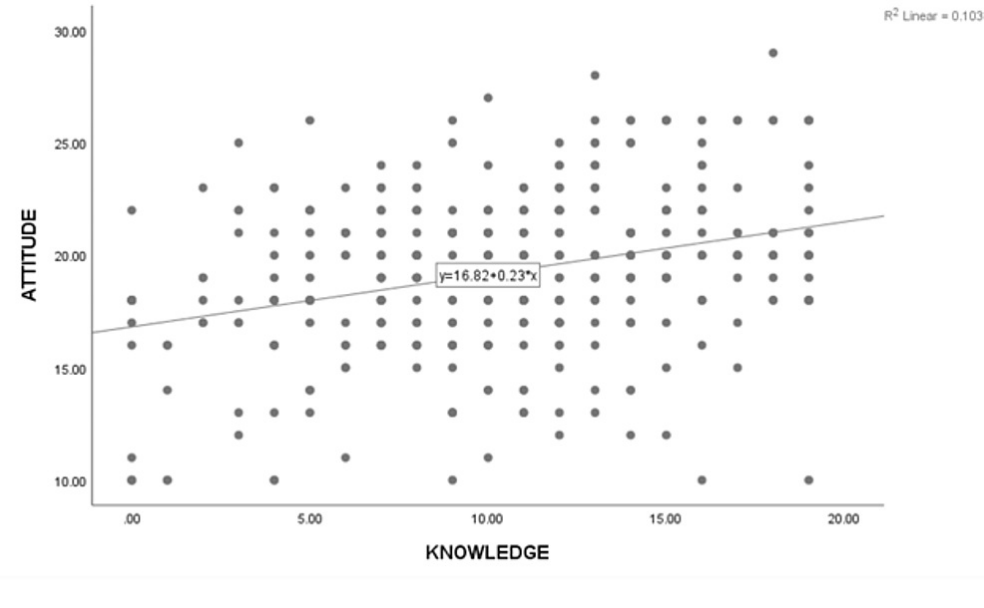
Association between knowledge level and attitude scores

Moreover, there is a positive correlation between knowledge level and attitude score (r = 0.321, p < 0.001). In other words, as participants' knowledge levels increase, their attitudes become more positive (Figure 1).

## Discussion

The demographics of the surveyed population play a pivotal role in understanding the varying knowledge and attitudes toward thyroid health in Jeddah, Saudi Arabia. Our results showed that 72.5% of respondents were female and 27.5% male. This agrees with a previous study [6], highlighting that women are often more proactive in seeking health information.

The age distribution of the surveyed population, with a median age of 30 years and an IQR of 21, mirrors the youthfulness and diversity identified in other studies [5]. However, there is a controversy between our results and that of another study conducted in Riyadh [14], as most of the participants are above 45 years (47%). This difference may be because all their participants already have hypothyroidism, which occurs more in older age. These findings emphasize the importance of tailoring health messages according to the distinct needs and preferences of different age groups.

The high educational attainment of 78.1% of participants, with a bachelor's degree or higher, aligns with previous research [1,5]. This is generally seen as a positive sign as higher education is often associated with better health information-seeking behavior. However, 21.9% of participants with a high school education or below present a challenge as language preference and health literacy cause negative health information-seeking experiences, as recognized in the study by Shu et al. [15]. To address this disparity, it is crucial to design accessible, simple educational materials to ensure inclusivity.

Our results showed that 14.8% of our participants were previously diagnosed with thyroid disease. Other studies conducted in Saudi Arabia [16,17] showed that the respondents had thyroid disorders representing 20.09% and 20.8%, respectively, which were higher than our results which may be because of the difference in the study population. Hypothyroidism is the most common thyroid disorder in our study and others. These findings are in line with the findings of Almousa et al. [9] and Ali et al. [18]. This underlines the importance of targeted educational efforts and awareness campaigns, particularly for the most prevalent thyroid condition, which is hypothyroidism.

There is a knowledge gap among our participants, with more than 50% of participants categorized as having "Poor" knowledge, which agrees with the findings of Alyahya et al. [5]. Moreover, according to a study done in India, most of the participants had inadequate knowledge and misconceptions about the thyroid gland and associated disorders [19]. This suggests that a substantial portion of the surveyed individuals may lack essential knowledge about thyroid diseases. Moreover, the distribution of knowledge levels from "Poor" to "Fair" in nearly 80% of participants aligns with the recognition of a need for tailored education efforts and interventions. Limited awareness, education, and cultural beliefs coupled with the complexity of thyroid disorders and the overlap of the symptoms with other health problems may lead to poor knowledge.

On the contrary, a study was done in Riyadh [16] that shows a different level of knowledge where 57% of the participants had a good level of knowledge. This difference may be because they classify knowledge into poor (score < 9/16) or good (> 9/16) only, but we classified knowledge into poor (< 10/19), fair (10-15/19), and good knowledge (> 15/19).

Not only do the general population have poor knowledge of thyroid disorders but also physicians as in the study by Askari et al. [20] conducted on general practitioners in Iran, which showed that their mean knowledge score was 39.9%.

The gaps in knowledge among respondents regarding the impact of pregnancy and the postpartum period, medications such as amiodarone, and the role of soy food in thyroid diseases concur with previous research [21]. This consensus emphasizes the pressing need for focused educational interventions to address these specific areas of misunderstanding and misinformation, ensuring that respondents have access to accurate and comprehensive information. Pregnancy is a very important issue as thyroid disease in pregnancy may lead to miscarriage, preterm birth, or fetal death.

These results agree with the study of Alyahya et al. [5], who found a knowledge gap among participants about the role of amiodarone and eating soya as risk factors for thyroid diseases. This is also following another study [22] done on hypothyroid patients where 54.6% did not avoid eating cabbage, cauliflower, and soya. However, in the study of Almousa et al. [9], 74.6% of participants thought that cabbage, cauliflower, and soy products should be avoided in hypothyroidism. This higher percentage could reflect greater awareness or different health education emphasizing the potential goitrogenic effect of these foods.

Our results showed that participants are unaware of GIT symptoms of thyroid disorders, which may delay the diagnosis.

The differing perceptions of the significance of family history as a risk factor compared to the expressed concern about the seriousness of thyroid diseases can be linked to the work of Li et al. [23]. Their findings, similar to ours, suggest that while respondents may not perceive family history as a risk factor, they are highly concerned about the severity of thyroid conditions. This disparity between knowledge and fear warrants additional investigation and education to mitigate misconceptions and reduce undue anxiety.

The prevailing negative attitudes toward thyroid health among the participants, as noted in our study, are in line with the findings of the study of Dew et al. [24]. However, an opposing view presented in another study [25] highlights a more neutral attitude in certain populations. These conflicting viewpoints underline the importance of addressing and correcting negative perceptions to promote positive health behaviors and call for further research to explore the factors contributing to these attitudes.

The association between knowledge level and sociodemographic data reveals a significant influence of educational level and employment status on knowledge levels. This is well-documented in the work of Dew et al. [25]. However, the revelation that participants who are students exhibit the highest knowledge levels aligns with findings by Askari et al. [20]. These observations underscore the importance of personalized education strategies for different educational backgrounds and demographics. Other studies in Saudi Arabia [1] showed that there is a correlation between gender education and knowledge levels. This is contrary to other studies [16,17], wherein gender and level of education had no significant impact on overall knowledge.

The significant association between a history of thyroid disease diagnosis and attitude level found in our study is corroborated by the work of Uslar et al. [25]. This suggests that individuals with personal experience of thyroid disease tend to have more positive attitudes, highlighting the potential of lived experiences in shaping perceptions.

The positive correlation between knowledge level and attitude score, which we identify, is consistent with the findings of Li et al. [24]. This underscores the pivotal role of knowledge in shaping attitudes and subsequently promoting healthier behaviors.

The diverse sources contributing to respondents' knowledge about thyroid diseases, including physicians, social media, and combined sources, resonate with existing literature. This diversity emphasizes the importance of ensuring that healthcare professionals provide accessible and understandable information, while information from social media should be monitored for accuracy and reliability. In the study of Rani et al. [26], the source of information is mainly from the neighbors and only 13.3% from doctors.

In summary, the agreement and dissent found in the existing literature provide a comprehensive understanding of the surveyed population's demographics and their implications for knowledge and attitudes related to thyroid health. These insights underscore the complexity of the subject and the need for tailored approaches to address varying knowledge levels and attitudes among different demographic groups.

Study limitations

The data collected in our study is based on self-reports, which can be subject to recall bias or social desirability bias. Respondents might provide answers that they think are expected rather than their true beliefs or behaviors. Moreover, a cross-sectional study design provides a snapshot of knowledge and attitudes at a specific point in time. It does not allow for the assessment of changes over time or the establishment of causal relationships.

## Conclusions

Our results highlight that higher knowledge levels parallel education attainment. Moreover, the attitude of the participants is correlated with their knowledge level. This correlation reinforces the pivotal role of knowledge in shaping healthier attitudes and behaviors. The diverse sources of knowledge, including healthcare professionals and social media, underscore the importance of accessible and accurate information and the need to monitor online health information. Medical professionals offer accurate personalized advice, but they can be limited by accessibility and communication barriers. While the internet can provide easy access to various information, its quality may be inconsistent.

By addressing these challenges and building upon the insights from this study, we can work toward enhancing the awareness and understanding of thyroid diseases through organizing community-based awareness campaigns and workshops, utilizing technology for remote education, and addressing the misconceptions about hypothyroidism. Overall, our results can ultimately improve the overall health and well-being of the population in Jeddah and beyond.
